# Hour-1 Sepsis Bundle: Updated Evidence

**DOI:** 10.3390/jcm15156049

**Published:** 2026-08-04

**Authors:** Gennaro De Pascale, Salvatore Lucio Cutuli, Simone Carelli, Irene Cisterna, Pierluigi Del Vecchio, Emanuele Oscar Franchini, Flavia Lucia Grilli, Gianmarco Lombardi, Altea Palladini, Andrea Tagliamonte, Eloisa Sofia Tanzarella, Guru Tudimella, Luca Montini, Domenico Luca Grieco, Massimo Antonelli

**Affiliations:** 1 Department of Biotechnologies, Intensive Care and Perioperative Medicine, Catholic University of the Sacred Heart, 00168 Rome, Italy; gennaro.depascalemd@gmail.com (G.D.P.); emanuele.franchini01@gmail.com (E.O.F.); flavia.grilli@yahoo.it (F.L.G.); altea.palladini@gmail.com (A.P.); andrea.tagliamonte@guest.policlinicogemelli.it (A.T.); gurutudimella@gmail.com (G.T.); luca.montini@policlinicogemelli.it (L.M.); domenicoluca.grieco@policlinicogemelli.it (D.L.G.); massimo.antonelli@policlinicogemelli.it (M.A.); 2 Department of Emergency Sciences, Anaesthesiology and Intensive Care Medicine, Fondazione Policlinico Universitario A. Gemelli IRCCS, 00168 Rome, Italy; simone.carelli@policlinicogemelli.it (S.C.); cisterna.irene@gmail.com (I.C.); gianmarco.lombardi@policlinicogemelli.it (G.L.); eloisasofia.tanzarella@policlinicogemelli.it (E.S.T.); 3 Department of Medical and Surgical Sciences, Fondazione Policlinico Universitario A. Gemelli IRCCS, 00168 Rome, Italy; pierluigi.delvecchio@policlinicogemelli.it

**Keywords:** sepsis, septic shock, bundle, intensive care, critical care

## Abstract

Sepsis and septic shock remain leading causes of disability, mortality and healthcare utilization worldwide. Increasing evidence has established sepsis as a time-dependent emergency in which delays in diagnosis and treatment are associated with worsening organ dysfunction and increased mortality. To standardize early management, the Surviving Sepsis Campaign (SSC) developed evidence-based care bundles, culminating in the current Hour-1 Bundle, which emphasizes rapid implementation of key diagnostic and therapeutic interventions. This narrative review critically examines the scientific rationale, clinical evidence, implementation challenges, and future perspectives surrounding the SSC Hour-1 Bundle. Lactate remains a valuable marker of illness severity and treatment response, although its interpretation requires consideration of multiple non-hypoperfusion-related mechanisms. Blood cultures represent the microbiological gold standard, while emerging rapid diagnostic technologies are transforming pathogen identification and antimicrobial stewardship. Early effective antimicrobial therapy remains strongly associated with improved outcomes, although balancing prompt treatment with antimicrobial stewardship remains challenging. Fluid resuscitation strategies have evolved from fixed-volume approaches toward individualized assessment of fluid responsiveness and tolerance, whereas early vasopressor initiation is increasingly recognized as complementary to fluid administration. Although observational studies frequently associate bundle adherence with improved outcomes, real-world compliance remains variable and evidence supporting strict one-hour completion targets remains heterogeneous. Future developments are expected to move beyond standardized protocols toward personalized sepsis management integrating rapid diagnostics, immune phenotyping, advanced haemodynamic monitoring, and artificial intelligence-driven clinical decision support. These innovations may enable more precise therapeutic strategies while preserving the fundamental principles of sepsis management, that remain the cornerstone of contemporary sepsis care.

## 1. Introduction

Sepsis and septic shock remain major causes of morbidity, mortality and healthcare utilization worldwide [1], accounting for a substantial proportion of intensive care unit (ICU) admissions [2]. Despite significant advances in critical care, sepsis continues to be associated with high short- and long-term mortality, prolonged hospitalization, and considerable economic burden. The heterogeneous host response to infection, ranging from excessive inflammation to profound immune dysfunction, contributes to the complexity of diagnosis and management and remains a major challenge for clinicians [3,4].

Over the last two decades, accumulating evidence has demonstrated that sepsis is a time-dependent medical emergency, as delays in recognition and treatment have consistently been associated with worsening organ dysfunction and increased mortality [2,5,6]. Early administration of appropriate antimicrobials [7,8], timely and effective control of source of infection [6], prompt implementation of supportive measures including adequate haemodynamic resuscitation, have therefore become central components of modern sepsis care [2]. This evidence has driven the development of standardized care pathways aimed at improving the consistency and timeliness of clinical interventions.

The Surviving Sepsis Campaign (SSC) introduced sepsis bundles to translate evidence-based recommendations into bedside practice and facilitate quality improvement initiatives [2]. Initially structured as the 24 h bundle in 2004 [9], followed by the 6 h and 3 h bundles in 2012 [10], these recommendations evolved into the Hour-1 Bundle in 2018 [11], emphasizing the prompt implementation of key diagnostic and therapeutic interventions following recognition of sepsis or septic shock. Although the Hour-1 Bundle [11] remains a practical framework for early sepsis management, the 2026 SSC guidelines [2] formulate evidence-based recommendations for each intervention individually, acknowledging differences in the strength and certainty of the supporting evidence. Accordingly, the current guidelines continue to recognize sepsis as a medical emergency requiring immediate recognition and treatment, while placing greater emphasis on clinical judgement and individualized management rather than rigid adherence to a universal one-hour timeline for every bundle component.

This review critically examines the components of the SSC Hour-1 Bundle [11], including lactate measurement, microbiological investigations, antimicrobial therapy, fluid resuscitation, and vasopressor support. Unlike the 2026 SSC guidelines [2], which provide evidence-based recommendations for clinical practice, this narrative review is not intended to replace them. Instead, it critically appraises the rationale underpinning current recommendations, discusses areas of ongoing uncertainty and controversy, integrates emerging evidence relevant to clinical practice, and highlights knowledge gaps that may inform future research and subsequent guideline updates. Although the Hour-1 Bundle [11] provides the conceptual framework for this review, selected complementary topics—including source control, immune profiling, immunomodulation, and artificial intelligence—are discussed insofar as they directly influence the delivery, optimization, and future evolution of early sepsis management. Finally, we examine real-world implementation, adherence, clinical performance, ongoing controversies, and future directions that may shape the next generation of sepsis care.

## 2. Literature Search Strategy

This narrative review was based on a non-systematic literature search conducted using PubMed/MEDLINE and Scopus. Relevant articles published in English up to 30 June 2026 were identified using combinations of the terms “sepsis”, “septic shock”, “Surviving Sepsis Campaign”, “Hour-1 Bundle”, “lactate”, “fluid resuscitation”, “vasopressors”, “antimicrobial therapy”, “source control”, and “implementation”. Priority was given to the 2026 SSC guidelines [2], randomized controlled trials, meta-analyses, high-quality observational studies, and landmark publications considered relevant to the topics discussed. As this is a narrative review, no predefined study protocol, formal study selection process, or standardized assessment of methodological quality or risk of bias was performed.

## 3. Hour-1 Bundle

The SSC Hour-1 Bundle [11] integrates five evidence-based interventions that should be initiated promptly after recognition of sepsis or septic shock: lactate measurement, blood culture acquisition before antimicrobial therapy whenever feasible, early administration of broad-spectrum antimicrobials, initial crystalloid resuscitation, and timely vasopressor support.

**Figure 1:**
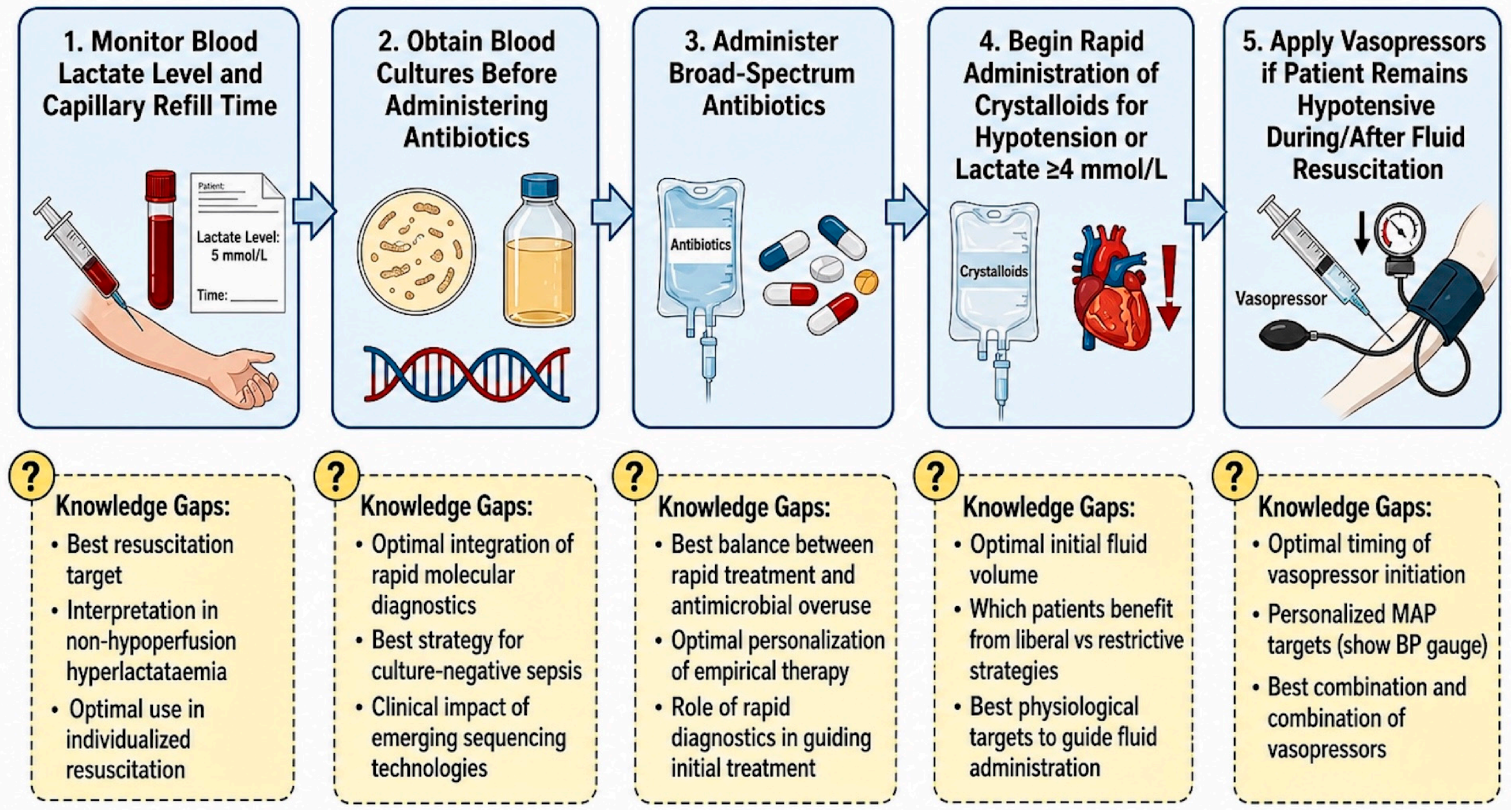
Surviving Sepsis Campaign Hour-1 Bundle: current recommendations and remaining knowledge gaps.

In the following sections, each bundle component is discussed by critically summarizing the available evidence supporting its implementation, highlighting the major controversies, and identifying the principal knowledge gaps that continue to limit optimal application in clinical practice (Table 1 and Figure 1).

### 3.1. Measure Blood Lactate Level (And Re-Measure if Elevated)

Blood lactate level has been considered one of the hallmarks of septic shock, and contributes to the diagnosis of this condition when concentrations are ≥2 mmol/L [12]. Although elevated lactate concentration has been associated with altered tissue perfusion and oxygen delivery to consumption (DO_2_-VO_2_) mismatch, the pathophysiological mechanisms underlying hyperlactataemia in sepsis remain incompletely understood [13]. During sepsis and septic shock, increased lactate production resulting from anaerobic glycolysis alone cannot fully explain the elevated concentrations observed in many patients [14]. Indeed, a growing body of evidence suggests that hyperlactatemia may occur in the absence of overt tissue hypoxia, mitochondrial dysfunction or DO_2_-VO_2_ mismatch. In this setting, the physiological stress response to sepsis may increase both carbohydrate metabolism and catecholamines release, each of which may independently contribute to lactate production [14]. These mechanisms promote pyruvate generation and subsequent lactate formation, with excess lactate being recycled into glucose through the Cori Cycle [15]. Furthermore, catecholamines stimulate Na^+^/K^+^- ATPase activity, thereby directly enhancing lactate production [16].

Approximately 1500 mmol of lactate are produced daily by organ metabolism, and are subsequently catabolised by the liver and kidneys [17]. Normal blood lactate concentration is approximately 1.0 ± 0.5 mmol/L under physiological conditions, while a concentration ≥2 mmol/L was associated with worse patient-related clinical outcomes [2]. Nevertheless, several studies suggest that adverse outcomes may be associated with lactate concentrations well below this threshold. Indeed, lactate concentrations >0.75 mmol/L at ICU admission have been associated with increased mortality regardless of the reason for admission, suggesting that the transition from physiological to pathological lactate metabolism may occur before conventional upper limits are exceeded [18]. In a large study by Casserly et al., lactate concentrations >4 mmol/L accompanied by hypotension were associated with increased mortality, and this risk persisted regardless of lactate trajectory over the following hours [19].

Serial lactate measurements also provide important prognostic information, as persistently elevated or rising lactate during the first 24 h of sepsis management are consistently associated with increased mortality [20]. In addition, lactate kinetics may be used to guide resuscitation, as reported by Nguyen et al. [21] with an 11% reduction in the likelihood of mortality for every 10% decrease in lactate concentration. Similarly, Jansen et al. [22] demonstrated in a multicentre randomised controlled trial that an early lactate-guided resuscitation strategy, targeting a reduction of more than 20% within the first 8 h of ICU admission, reduced both ICU and in-hospital mortality. Collectively, these findings support the concept that improvement in metabolic status represents a key target of resuscitation in septic shock. Conversely, large randomised trials have demonstrated that resuscitation strategies guided by alternative markers of tissue hypoperfusion, such as capillary refill time (CRT), achieve outcomes comparable to those of serial lactate-guided approaches [23,24]. In the ANDROMEDA-SHOCK trial [23], CRT-guided resuscitation targeting normalization (CRT ≤ 3 s) resulted in less organ dysfunction at 72 h, with a trend towards lower 28-day mortality compared with lactate-guided resuscitation. Building on these findings, the recent ANDROMEDA-SHOCK-2 trial [24] demonstrated that a personalised haemodynamic strategy targeting CRT normalisation improved a hierarchical composite endpoint of mortality, duration of vital organ support and hospital length of stay, compared with the standard of care. These findings supported CRT as a pragmatic bedside target for guiding early septic shock resuscitation. Importantly, the choice of resuscitation targets may be more relevant than the monitoring technique itself. In the recent EVERDAC trial [25], an initial strategy based on non-invasive oscillometric blood pressure monitoring was non-inferior to early arterial catheterisation with respect to 28-day mortality, while avoiding arterial catheter placement in approximately 85% of patients, thereby preventing arterial catheter-related complications. These findings suggest that routine early arterial catheterisation may not be necessary in all patients with septic shock and support an individualised approach to haemodynamic monitoring based on illness severity and the need for advanced haemodynamic assessment.

Strategies aimed at achieving lactate clearance within the first hour of sepsis or septic shock recognition should be individualised, as multiple causes of hyperlactatemia may coexist. In this context, enhanced metabolic activity favouring pyruvate-to-lactate conversion, such as seizures or intense exercise, as well as impaired tissue perfusion resulting from cardiac arrest or carbon monoxide poisoning, may lead to elevated lactate concentrations [26]. Additionally, clinicians should be aware of other clinical conditions associated with hyperlactatemia, that include diabetes [27], malignancy [28], alcohol use disorder, thiamine deficiency [29], and mitochondrial dysfunction [30]. Consequently, differential diagnosis and comprehensive clinical assessment remain essential when interpreting hyperlactataemia, as emphasised by current guidelines [2].

### 3.2. Obtain Blood Cultures Before Administering Antibiotics

Blood cultures remain a cornerstone of early sepsis management because microbiological identification supports antimicrobial stewardship programmes by enabling targeted antimicrobial therapy and facilitating antimicrobial de-escalation. The 2026 SSC guidelines [2] strongly recommend obtaining blood cultures as soon as possible, ideally before antimicrobial therapy, while emphasizing that microbiological sampling should not result in clinically meaningful delays to antimicrobial administration, particularly in patients with septic shock. Despite remaining the microbiological gold standard, the diagnostic yield of blood culture in sepsis is often limited and heterogeneous, with positivity rates ranging from approximately 10–20% in unselected patients with to substantially higher rates in septic shock, endovascular infections, and infective endocarditis [31,32]. Culture sensitivity is influenced by multiple factors, including pathogen load, the source of infection, prior antimicrobial exposure, the timing of collection, and technical aspects of sample collection. Adequate blood volume remains one of the major determinants of diagnostic sensitivity, as initially demonstrated by Weinstein et al. [33] and subsequently confirmed by studies evaluating blood culture optimization strategies [34]. Inadequate antisepsis and suboptimal collection techniques may also increase contamination rates, leading to false-positive cultures, unnecessary antimicrobial exposure, and prolonged hospitalization [31,34].

Balancing rapid antimicrobial administration with optimal microbiological sampling remains a major challenge in sepsis care. Kumar et al. [35] demonstrated that delayed effective antimicrobial therapy in septic shock is associated with increased mortality for every hour of delay after the onset of hypotension [35]. Conversely, other research showed that even brief antimicrobial exposure significantly reduces blood culture positivity [36,37]. In the multicentre cohort study by Cheng et al. [37], blood culture positivity decreased from 31.4% before antibiotic administration to 19.4% afterward, corresponding to a 38% relative reduction in diagnostic yield after a median interval of 70 min.

Previous antimicrobial exposure remains one of the strongest predictors of culture-negative sepsis and reduced microbiological recovery [37,38]. Diagnostic reliability can be enhanced by optimising microbiological sampling through appropriate skin antisepsis, adequate blood volume collection, rapid transport to automated incubation systems, and early source-directed sampling (e.g., bronchoalveolar lavage in pneumonia), while simultaneously reducing contamination rates [31,34]. In this context, rapid diagnostic technologies are progressively reshaping sepsis microbiology [39]. Multiplex PCR platforms, MALDI-TOF mass spectrometry, rapid phenotypic susceptibility testing, and syndromic respiratory panels significantly reduce time to pathogen identification and resistance detection compared with conventional cultures [40]. Similarly, the FAST randomized clinical trial by Banerjee et al. showed that rapid antimicrobial susceptibility testing shortened time to antimicrobial optimization and increased stewardship-guided therapy in Gram-negative bloodstream infections, despite not meeting its primary endpoint [41]. Emerging metagenomic approaches, including plasma microbial cell-free DNA sequencing platforms such as the Karius test, may further expand diagnostic capabilities in culture-negative sepsis and invasive pulmonary infections, although their role in routine ICU practice remains to be fully established [42]. In severe respiratory infections, Virk et al. reported that rapid multiplex PCR panels were associated with faster antimicrobial escalation and earlier Gram-positive de-escalation, whereas Cano et al. demonstrated that protocolized stewardship strategies based on FilmArray testing facilitated earlier antimicrobial optimization in mechanically ventilated ICU patients [43,44].

### 3.3. Administer Broad-Spectrum Antibiotics

The 2026 SSC guidelines [2] distinguished between patients with septic shock, patients with definite or probable sepsis without shock, and patients with possible sepsis without shock. In patients with septic shock, immediate administration of broad-spectrum antimicrobial therapy remains a priority because delays are consistently associated with worse outcomes. Likewise, patients with definite or probable sepsis without shock should receive antimicrobial therapy as soon as possible, ideally within 1 h of recognition. By contrast, in patients with possible sepsis without shock, the guidelines recommend a rapid but focused diagnostic assessment before initiating antimicrobial therapy, provided this does not delay treatment beyond 3 h when infection remains the most likely diagnosis. This strategy seeks to balance the benefits of early antimicrobial therapy in patients at highest risk of deterioration with the principles of diagnostic and antimicrobial stewardship, minimizing unnecessary exposure to broad-spectrum agents while avoiding inappropriate treatment delays in patients with true sepsis. In addition, the 2026 SSC guidelines [2] introduce a new recommendation suggesting prehospital administration of antimicrobials in ambulances or air medical transport when the anticipated time to hospital-based medical evaluation exceeds 60 min.

Early administration of broad-spectrum antimicrobial therapy to provide empirical coverage of the most likely causative pathogen(s), together with effective source control, remains a cornerstone of the management of sepsis and septic shock. Over the past two decades, extensive evidence has demonstrated that delays in effective antimicrobial therapy and source control are associated with increased mortality, supporting the 2026 SSC [2] recommendation for early antimicrobial administration in patients with sepsis or septic shock [45]. This approach may involve either initiating a new antimicrobial regimen or broadening the spectrum of ongoing treatment when clinical deterioration suggests treatment failure or progression of the underlying infection [2]. Despite this evidence [46], optimization of empirical antimicrobial therapy continues to represent a major challenge in routine clinical practice.

Adequate exposure to antimicrobials is challenged by the profound pharmacokinetic alterations induced by sepsis, resulting from both organ dysfunction (particularly hemodynamic dysfunction and acute kidney injury) and therapeutic interventions such as aggressive fluid resuscitation [47,48]. During the early hyperdynamic phase of sepsis, systemic inflammation, endothelial dysfunction, increased capillary permeability and capillary leak syndrome, together with large-volume fluid administration, expand the extracellular fluid compartment, leading to an increased volume of distribution (Vd). Because hydrophilic antimicrobials, including β-lactams and aminoglycosides, are predominantly distributed within the extracellular space, this increase in Vd reduces initial plasma drug concentrations after the first dose, increasing the risk of early target non-attainment [47,49]. In parallel, the hyperdynamic circulatory state frequently observed during the initial phase of sepsis may result in augmented renal clearance (ARC), particularly in younger patients and those with preserved renal function, thereby accelerating the elimination of renally cleared antibiotics and further compromising adequate drug exposure despite standard dosing regimens [47,48]. Consequently, an adequate loading dose should be administered early to rapidly achieve therapeutic concentrations, whereas subsequent maintenance dosing should be individualized according to renal function, the presence of ARC, extracorporeal organ support when applicable, and therapeutic drug monitoring whenever available [47,48,49].

Moreover, multiple audits continue to report substantial rates of inappropriate initial antimicrobial therapy [50], that may reflect unrecognized risk factors for multidrug-resistant pathogens (MDR) and delayed recognition of sepsis [51].

Decisions regarding empirical coverage for MDR pathogens and invasive fungal infections remain controversial. The need for MDR coverage depends largely on patient-related risk factors, including colonization or previous infection with MDR pathogens, prolonged exposure to broad-spectrum antimicrobials, and extended hospitalization [2]. Accordingly, active surveillance cultures may help to identify high-risk patients and guide empirical treatment decisions, particularly in lower respiratory tract infections [52]. Similarly, careful assessment of host-related risk factors is essential when considering empirical antifungal therapy. Current evidence does not demonstrate mortality benefit associated with empiric antifungal therapy in critically ill, non-neutropenic patients [53]. Nevertheless, specific clinical features, including immunosuppression, prolonged antimicrobial exposure, and intra-abdominal source of infection, may identify patients at increased risk of invasive fungal disease, and therefore support consideration of early antifungal coverage [54,55].

However, these potentially life-saving interventions should be integrated within a structured decision-making framework. Selection of empirical therapy should be guided by the suspected site of infection, local epidemiology, severity of illness, host-related risk factors, and the pharmacokinetics/pharmacodynamic (PK/PD) characteristics of antimicrobials. Equally important is balancing the need for early effective treatment with principles of antimicrobial stewardship to minimise unnecessary antimicrobial exposure and limit the emergence of MDR [56,57]. Additionally, optimising antimicrobial dosing represents a further challenge in critically ill patients, in whom profound pathophysiological alterations frequently result in subtherapeutic drug exposure. In this setting, therapeutic drug monitoring may therefore represent a valuable tool to maximise antimicrobial exposure whilst reducing the risk of antimicrobial toxicity [51].

With regard to the timing of antimicrobial administration, the recommendations remain largely unchanged from the previous guidelines [58]. A strong recommendation is maintained for initiating antimicrobial therapy within 1 h of recognition in patients with septic shock, when the diagnosis is definite, probable or possible, and in patients without shock when sepsis is definite or probable. In patients without shock, in whom sepsis is considered possible, antimicrobial therapy should be initiated within 3 h following a rapid clinical assessment. In addition, the 2026 SSC guidelines [2] introduce a new recommendation suggesting pre-hospital administration of antimicrobials in ambulances or air medical transport when the anticipated time to hospital-based medical evaluation exceeds 60 min [2].

### 3.4. Begin Rapid Administration of Crystalloids for Hypotension or Lactate ≥ 4 mmol/L

Early intravenous fluid administration remains a cornerstone of the initial management of septic shock, particularly in patients presenting with hypotension or hyperlactataemia. The physiological rationale lies in the haemodynamic derangements that characterise early sepsis, where systemic vasodilation, increased capillary permeability, and relative hypovolemia reduce effective circulating volume and impair tissue perfusion. Prompt administration of intravenous balanced crystalloids aims to restore preload, augment cardiac output, and prevent progression to refractory circulatory failure [2].

The recommendation to administer an initial fluid bolus of at least 30 mL/kg of crystalloid within the first 3 h (retained in the 2026 SSC guidelines [2] as a conditional recommendation based on low-certainty evidence) originates from observational studies linking early fluid administration with reduced mortality and more rapid shock resolution [2,59]. Cohort studies have suggested that receipt of 30 mL/kg within 3 h is associated with lower hospital mortality and reduced need for mechanical ventilation, with timing of administration emerging as an independent determinant of outcome [60].

However, more recent randomised trials have failed to demonstrate a mortality benefit for liberal fluid administration compared with restrictive strategies, reporting no significant differences in 90-day mortality between the two approaches [61,62]. These findings suggest that a fixed weight-based resuscitation strategy may result in overtreatment of a substantial proportion of patients, reflecting the marked inter-individual variability in haemodynamic response to fluid loading. This variability is explained by the Frank–Starling relationship, which describes the interplay between preload and stroke volume up to a physiological plateau. Patients with septic shock may occupy different positions along this curve: some remain on the ascending portion and demonstrate a meaningful increase in cardiac output following fluid administration, whereas others are already on the plateau portion, where additional fluid provides little haemodynamic benefit despite increasing cardiac filling pressures.

Consequently, the 30 mL/kg threshold should be viewed as an initial guide rather than a mandatory fixed-volume target to be administered indiscriminately to every patient. Individuals at increased risk of fluid overload, such as those with acute heart failure or end-stage kidney disease requiring dialysis, require careful clinical reassessment, integration of dynamic measures of fluid responsiveness when appropriate, and continuous evaluation of the balance between tissue perfusion and the risk of fluid accumulation. Accordingly, subsequent fluid administration should be individualized on the basis of repeated clinical assessment and fluid responsiveness rather than dictated solely by the initial recommended volume [2,63,64].

Fluid responsiveness is generally defined as an increase in cardiac output of more than 10–15%-depending on the haemodynamic monitoring method-following preload augmentation. Since only approximately 50% of haemodynamically unstable patients are fluid responders, indiscriminate fluid administration is unlikely to provide benefit and may expose patients to harm. Dynamic assessments—which include passive leg raising, pulse pressure variation, stroke volume variation, end-expiratory occlusion, and mini-fluid challenges (100 mL of crystalloids [65])—have demonstrated greater predictive accuracy than static haemodynamic variables, and should be used whenever feasible, according to ventilatory status and monitoring tools available [64,66]. Importantly, an increase in cardiac output does not necessarily translate into improved tissue oxygen consumption. Markers of DO_2_ and VO_2_, including lactate clearance and the veno-arterial gap of CO_2_ partial pressure over arterio-venous O_2_ content ratio are more reliable markers of O_2_ delivery and utilization coupling [67]. Equally important is the concept of fluid tolerance, defined as the patient’s capacity to receive additional fluids without developing clinically significant congestion or organ dysfunction. Assessment of fluid tolerance should therefore complement evaluation of fluid responsiveness in every resuscitation decision [68].

These principles underpin the contemporary ROSE model of fluid stewardship [69], which conceptualises fluid therapy as a dynamic process encompassing four sequential phases: Resuscitation, Optimisation, Stabilisation, and Evacuation (de-resuscitation). During the resuscitation phase, fluids are administered to rapidly restore tissue perfusion and haemodynamic stability. Subsequent optimisation aims to individualise fluid administration according to haemodynamic response and perfusion targets, whilst the stabilisation phase focuses on maintaining organ perfusion and avoiding unnecessary fluid accumulation. Finally, the evacuation phase seeks to achieve a neutral or negative fluid balance through spontaneous diuresis, diuretic therapy, or extracorporeal fluid removal when appropriate [69].

Current evidence therefore supports a phase-based approach to fluid management in septic shock. During the initial resuscitation phase, prompt administration of intravenous crystalloids remains appropriate in patients with sepsis-induced hypoperfusion or septic shock to restore effective circulating volume and improve tissue perfusion. Once the initial resuscitation has been completed, however, further fluid administration should no longer follow predetermined volume thresholds but instead be guided by repeated clinical assessment, dynamic measures of fluid responsiveness, and evaluation of fluid tolerance. Following hemodynamic stabilisation and control of shock, attention should shift towards avoiding persistent positive fluid balance and, whenever clinically appropriate, initiating active fluid removal (de-resuscitation). Although early fluid administration is beneficial when targeted to patients with evidence of hypoperfusion, randomized trials have not demonstrated superiority of liberal over restrictive fluid strategies beyond the initial resuscitation phase. Accordingly, the 2026 SSC guidelines support an individualized approach to fluid therapy throughout the course of critical illness rather than indiscriminate liberal fluid administration [2,61]. Several studies have demonstrated that higher cumulative fluid balance is independently associated with increased mortality among critically ill patients with sepsis and septic shock [70,71]. Excess fluid contributes to interstitial oedema, impaired gas exchange, and multiple organ dysfunction, particularly affecting pulmonary and renal function. For this reason, the current SSC guidelines recommend active fluid removal following completion of the initial resuscitation phase whenever clinically appropriate [2,61], to mitigate the harmful consequences of fluid accumulation [2,59].

### 3.5. Apply Vasopressors if the Patient Remains Hypotensive During or After Fluid Resuscitation

Vasopressor support is recommended when hypotension persists despite adequate fluid resuscitation. In patients with severe haemodynamic instability, however, norepinephrine should be initiated early and should not be delayed until completion of a predefined fluid volume [2,11]. Norepinephrine remains the first-line vasopressor. Its predominant α1-adrenergic activity restores systemic vascular resistances and recruits unstressed venous volume, thereby increasing venous return and, in preload-responsive patients, cardiac output. In addition, modest β1-adrenergic stimulation may improve myocardial contractility and contribute to haemodynamic stabilization [72,73,74]. However, the optimal timing of norepinephrine initiation remains an area of ongoing investigation. The CENSER trial showed that early low-dose norepinephrine improved the management of shock control within six hours, reduced the incidence of cardiogenic pulmonary oedema and new-onset arrhythmias [75]. Similarly, observational propensity-matched studies have associated very early norepinephrine administration with lower mortality and reduced cumulative fluid balance [76]. In contrast, the CLOVERS trial [62] found no significant differences in mortality or organ-support-free days between a restrictive fluid strategy incorporating earlier vasopressor use and a more liberal fluid approach. These findings have recently been reinforced by the ARISE FLUIDS trial [77], which randomised patients with early septic shock to a strategy of restricted fluids with earlier vasopressor initiation or greater fluid volumes with later vasopressor therapy. Despite achieving a separation of more than 1 L of intravenous fluid during the first 24 h and earlier vasopressor initiation (median 0.4 vs. 1.4 h), no differences were observed in days alive and out of hospital at day 90, neither in other secondary outcomes including mortality, organ support requirements or renal replacement therapy. However, the restrictive fluid/early vasopressor strategy was associated with a significantly lower incidence of pulmonary oedema, supporting the safety of earlier vasopressor initiation while suggesting that routine administration of large fluid volumes during early resuscitation may not be necessary for all patients. These findings support the concept that fluids and vasopressors should be considered complementary rather than mutually exclusive interventions. While fluids primarily increase stressed volume and preload, vasopressors restore vascular tone and redistribute venous capacitance [61,72]. Accordingly, haemodynamic resuscitation should be individualized by integrating early vasopressor therapy with judicious fluid administration according to the patient’s physiological response.

To facilitate early hemodynamic stabilization, current 2026 SSC guidelines [2] support initiation of vasopressors through a peripheral venous catheter when central venous access is not immediately available, rather than delaying treatment until central access has been secured. Recent meta-analyses have reported a low incidence of local complications associated with peripheral vasopressor administration when delivered within structured safety protocols that include appropriate catheter selection, frequent monitoring for extravasation, and predefined limits on infusion duration [78]. Nevertheless, available evidence remains insufficient to establish optimal catheter calibre and length, insertion site, or maximal safe infusion dose.

Current guidelines recommend an initial mean arterial pressure (MAP) target of approximately 65 mmHg for most patients with septic shock [2]. In patients aged 65 years or older, a target MAP of 60–65 mmHg may be considered in preference to higher values [2]. The SEPSISPAM trial [79] demonstrated that targeting a MAP of 80–85 mmHg did not improve survival and was associated with higher incidence of atrial fibrillation compared with 65–70 mmHg. Similarly, the 65-trial [80] showed that permissive hypotension, targeting a MAP of 60–65 mmHg, reduced vasopressor exposure without adversely affecting mortality in older patients. However, MAP targets should be individualised according to patient characteristics and evidence of organ perfusion. Notably, in the subgroup of patients with chronic hypertension enrolled in the SEPSISPAM, higher MAP targets were associated with a reduced need for renal replacement therapy [79].

Escalating norepinephrine requirements should prompt comprehensive haemodynamic reassessment to identify persistent preload responsiveness, ongoing vasodilation, or concomitant myocardial dysfunction [73]. Additional fluid administration, inotropic support, or adjunct vasopressor therapy should be considered according to the underlying haemodynamic phenotype [73]. Contemporary management of septic shock increasingly favours a multimodal vasopressor strategy aimed at reducing excessive catecholamine exposure through the early introduction of agents with complementary mechanisms of action. Vasopressin is the preferred second-line vasopressor and may reduce the incidence of atrial fibrillation whilst potentially decreasing the need for renal replacement therapy, particularly when introduced at lower norepinephrine doses, although no consistent mortality benefit has been demonstrated [2,81,82]. Epinephrine is recommended when an additional vasopressor effect is required or when vasopressin is unavailable [2]. Angiotensin II represents a further therapeutic option for selected patients with refractory vasodilatory shock. Although biomarker-guided approaches based on renin concentrations have generated considerable interest, clinical evidence remains limited. In the ATHOS-3 trial [83], angiotensin II effectively increased MAP and reduced catecholamine requirements in patients with vasodilatory shock, although no improvement in mortality was observed as the study was not powered to detect survival differences.

Taken together, the available evidence suggests that early norepinephrine administration is safe, facilitates more rapid achievement of target arterial pressure, and may reduce cumulative fluid administration and fluid-related complications, including pulmonary oedema. However, despite these physiological advantages, current randomized evidence has not demonstrated a consistent reduction in mortality or other major patient-centred outcomes with strategies favouring earlier vasopressor initiation and more restrictive fluid administration compared with contemporary standard care. Therefore, the 2026 SSC guidelines support early norepinephrine as part of an individualized haemodynamic resuscitation strategy, integrated with careful assessment of tissue perfusion and fluid responsiveness, rather than as a universal replacement for initial fluid resuscitation [2].

## 4. Implementation, Compliance, and Real-World Performance of 1 h Bundle

Despite widespread endorsement, adherence to the Hour-1 bundle remains highly variable across healthcare systems and clinical settings. In a Japanese multicentre cohort study, approximately half of patients received all bundle components within one hour, and complete bundle adherence was associated with significantly lower in-hospital mortality [84]. Similarly, Evans et al. [5], in a multicentre paediatric study conducted in the United States before formal publication of the Hour-1 Bundle, reported complete adherence to an equivalent early sepsis bundle in only 24.9% of patients, with substantial variability between institutions. These findings highlight the persistent challenges associated with translating guideline recommendations into routine clinical practice.

Several factors contribute to incomplete bundle implementation. Diagnostic uncertainty remains one of the most important barriers, as sepsis frequently presents with non-specific or atypical clinical manifestations, particularly in older adults, immunocompromised individuals, and patients with multiple comorbidities [85]. Clinical concerns regarding potential adverse effects of treatment, including fluid overload, unnecessary antimicrobial exposure, or competing procedural priorities, may further delay implementation of bundle components [5,86]. Organisational factors also play a critical role. Emergency department overcrowding, staffing shortages, inadequate clinician education, absence of structured sepsis pathways, and limited access to diagnostic or therapeutic resources may all contribute to delayed sepsis recognition and treatment [87,88].

Although some studies reported that adherence to the Hour-1 bundle was independently associated with lower 90-day mortality [89], the overall evidence remains controversial. A large Korean observational study found no significant difference in hospital mortality between patients managed according to the Hour-1 bundle and those receiving bundle interventions within three or six hours [90]. Likewise, a stepped-wedge cluster-randomised trial conducted across 23 European emergency departments showed that improved bundle compliance achieved through implementation strategies was not accompanied by a reduction in mortality (12.1% vs. 12.6%) [91]. These conflicting findings may reflect important methodological limitations. Most of the available evidence derives from observational studies, making it difficult to distinguish the independent effects of bundle implementation from differences in patient characteristics, institutional performance, and overall quality of care. Institutions with higher bundle adherence often have established sepsis pathways, greater multidisciplinary expertise, and more mature quality-improvement programmes, all of which may independently contribute to better outcomes. Similarly, earlier recognition of sepsis may facilitate more timely overall management irrespective of bundle completion, while differences in case-mix, illness severity, treatment indication, and documentation practices may further confound the observed associations. Consequently, observational studies may overestimate the benefits directly attributable to bundle adherence. In addition, concerns have been raised regarding the possibility that strict time-based targets may inadvertently encourage inappropriate clinical decisions, including indiscriminate administration of broad-spectrum antimicrobials, unnecessary fluid resuscitation, or sepsis over-diagnosis. Taken together, these findings suggest that adherence to structured sepsis pathways remains an important quality-improvement measure. However, improvements in patient outcomes should be interpreted within the broader context of high-quality sepsis care rather than attributed solely to achievement of predefined bundle metrics.

## 5. Controversies and Future Directions

The 2026 SSC guidelines [2] represent the most comprehensive and evidence-based framework currently available for the management of sepsis and septic shock, whereas several important uncertainties remain. Future improvements in patient outcomes will likely depend not only on continued optimisation of bundle-based care but also on advances in source control, precision immunotherapy, and artificial intelligence-driven clinical decision support. Although the current recommendations continue to advocate time-sensitive bundled interventions to standardise early sepsis management and improve outcomes [2,92], source control remains a fundamental yet comparatively understudied component of care [93].

Current 2026 SSC guidelines [2] emphasise source control as a best-practice intervention and suggest that, whenever required, it should be performed as soon as medically, logistically, and safely feasible, ideally within six hours of diagnosis.

Source control encompasses all interventions aimed at eliminating the source of infection, reducing microbial burden, and restoring normal anatomy and function. Common examples include drainage of intra-abdominal abscesses, debridement of infected necrotic tissue, evacuation of empyema, and removal of infected vascular or orthopaedic devices. It also includes minimally invasive approaches, such as image-guided percutaneous drainage of infected collections and relief of biliary or urinary obstruction, whenever these provide effective elimination of the infectious focus. Antimicrobial therapy is an essential adjunct to source control but should not be considered a substitute for anatomical source control when an infected collection, devitalised tissue, obstructed system, or infected device persists. Despite broad consensus regarding its importance, considerable variability exists in the availability and delivery of source control interventions. In an international survey involving 1023 hospitals across 69 countries, only 87% of institutions reported round-the-clock access to surgical source control, whilst delays exceeding 12–24 h were reported in approximately 15% of centres, particularly outside intensive care settings [36]. Furthermore, patient-specific factors, infection characteristics, and local expertise frequently influence the choice between surgical and minimally invasive approaches. The optimal timing of source control remains uncertain. Premature intervention in inadequately resuscitated patients may worsen outcomes, whereas excessive delays may allow progression of organ dysfunction and ongoing microbial dissemination [94]. Reflecting these uncertainties, the 2026 SSC guidelines suggest that source control should be achieved as soon as medically, logistically, and safely feasible, ideally within 6 h of diagnosing sepsis or septic shock requiring source control [2]. In haemodynamically unstable patients, initial resuscitation should proceed in parallel with diagnostic evaluation and procedural planning, rather than unnecessarily delaying definitive source control. However, no validated physiological markers currently identify the optimal degree of haemodynamic stabilisation required before intervention, and prolonged attempts at medical stabilisation without controlling the infectious source are unlikely to be successful in patients with ongoing septic shock. Therefore, continuous multidisciplinary reassessment is essential to individualise the timing of source control according to the patient’s haemodynamic trajectory, the anatomical source of infection, procedural risk, and local expertise. At present, reliable physiological markers identifying when a patient is sufficiently stabilised to undergo source control are lacking and should represent a priority for future investigation.

Current sepsis guidelines [2] do not address the immune profiling into therapeutic decision-making, although patient outcomes are strongly influenced not only by pathogen-related factors but also by the host immune response.

Sepsis is now recognised as a highly heterogeneous syndrome characterised by dynamic and often simultaneous processes of hyperinflammation and immune suppression. Excessive inflammatory responses may contribute directly to tissue injury and organ dysfunction, whereas prolonged immune exhaustion increases susceptibility to secondary infections and poor long-term outcomes [95].

The PROVIDE study [96] represented a major step towards clinically meaningful immune stratification. Using serum ferritin concentrations and monocyte HLA-DR expression, investigators classified patients into three immunological phenotypes: macrophage activation-like syndrome (MALS), immunoparalysis, and an intermediate phenotype. Immune phenotype independently predicted 28-day mortality, 90-day mortality, and the occurrence of secondary infections. Patients with MALS exhibited profound hyperinflammation and the highest mortality, whilst patients with immunoparalysis experienced substantial mortality associated with severe immune dysfunction and increased vulnerability to nosocomial infection [96].

Building upon these observations, the ImmunoSep trial [97] explored a precision-medicine approach in which treatment was guided by immune phenotype. Administration of anakinra in patients with MALS and recombinant interferon-γ in patients with immunoparalysis resulted in significantly higher organ dysfunction improvement compared with placebo. In addition, reversal of sepsis-induced immune dysfunction occurred substantially more frequently among treated patients. These findings support the concept that immune heterogeneity in sepsis is not merely biologically relevant but may represent a therapeutic target. The lessons emerging from PROVIDE, ImmunoSep, and related studies converge on a central principle: future immunomodulatory trials should incorporate biomarker-guided patient stratification as a prerequisite for therapeutic success [4,96,97]. Whether phenotype-directed interventions, including anakinra for hyperinflammatory phenotypes and recombinant interferon-γ for immunoparalysis, can improve survival in larger and more diverse populations remains one of the most important unanswered questions in contemporary sepsis research.

Furthermore the 2026 SSC guidelines focused on quality improvement initiatives, defined as systematic efforts to improve evidence-based care processes and clinical outcomes through continuous monitoring, feedback, and organisational optimisation [2]. In this context, artificial intelligence (AI) and machine learning (ML) are increasingly being explored as decision-support tools with the potential to improve the early recognition of sepsis and facilitate timely implementation of evidence-based care bundles. However, the available evidence remains limited, and these technologies require robust prospective validation before they can be routinely integrated into clinical practice. Specifically, early warning systems based on real-time physiological monitoring [98] have demonstrated the ability to facilitate earlier recognition of sepsis and improve clinical outcomes in routine practice [99]. A recent meta-analysis [100] found that ML-based prediction systems were associated with greater reductions in sepsis-related mortality than traditional rule-based alert systems [95]. Beyond early detection, machine learning may also contribute to treatment optimisation. In a large cohort of more than 42,000 patients, Kalimouttou et al. [98] developed an ML-derived approach to identify the subset of guideline-based interventions most strongly associated with survival benefit. Adherence to the resulting six-item bundle was associated with lower mortality, whereas implementation of additional recommendations beyond those selected by the algorithm did not provide further benefit. Despite these promising results, substantial barriers remain before AI can be routinely integrated into sepsis management. Limited external validation, insufficient patient diversity within training datasets, concerns regarding algorithmic bias, clinician acceptance, and the absence of robust cost-effectiveness data continue to limit widespread adoption. Addressing these challenges will require close collaboration between clinicians, data scientists, healthcare organisations, and regulatory bodies. Ultimately, improving outcomes in sepsis will depend on the ability to combine timely evidence-based interventions with precision medicine approaches that recognise the biological heterogeneity of the syndrome. To achieve this goal, large collaborative clinical trials, robust implementation science, and refinement of the multidisciplinary framework will be required, alongside the evolution of the Surviving Sepsis Campaign in the next years to come.

## 6. Conclusions

The 2026 Surviving Sepsis Campaign (SSC) guidelines continue to provide the cornerstone for the early management of sepsis and septic shock by emphasizing the prompt implementation of evidence-based diagnostic and therapeutic interventions. Compared with the 2018 Hour-1 Bundle, the updated recommendations adopt an intervention-specific approach, highlighting the importance of clinical judgement and individualized patient management rather than a universal one-hour timeline. As the evidence base continues to evolve, further research is needed to strengthen recommendations for individual interventions and to better define the optimal timing and selection of therapies across different patient populations. Increasing evidence supports complementing early treatment with individualized assessment of perfusion, fluid responsiveness, infection risk, and host characteristics. Future studies should focus on optimizing source control strategies, validating biomarker-guided and immunophenotype-driven therapies, refining haemodynamic resuscitation targets, and evaluating the integration of artificial intelligence and machine learning into clinical decision support systems. These advances may further enhance personalized sepsis management and improve patient outcomes.

## Figures and Tables

**Table 1:** Current evidence and remaining knowledge gaps across the components of the Surviving Sepsis Campaign Hour-1 Bundle.

Bundle Component	Current Evidence	Knowledge Gap
*Measure blood lactate level (and re-measure if elevated)*	Marker of disease severity and prognosis. Serial measurements may help monitor response to resuscitation. Lactate clearance is associated with improved outcomes. CRT should be assessed as an adjunctive marker of peripheral perfusion, targeting a CRT ≤ 3 s.	Optimal resuscitation target (lactate clearance vs. alternative perfusion markers). Interpretation of hyperlactataemia in the absence of tissue hypoperfusion. Optimal integration of lactate and peripheral perfusion markers into individualized resuscitation strategies.
*Obtain blood cultures before administering antibiotics*	Blood cultures remain the microbiological gold standard. Sampling before antibiotics increases diagnostic yield. Supports targeted antimicrobial therapy and de-escalation.	Optimal integration of rapid molecular diagnostics. Best diagnostic strategy for culture-negative sepsis. Clinical impact of emerging sequencing technologies.
*Administer broad-spectrum antibiotics*	Early appropriate therapy reduces mortality, especially in septic shock. Empirical therapy should consider the suspected source of infection and risk factors for MDR pathogens. Antimicrobial stewardship remains essential.	Optimal balance between early treatment and avoidance of unnecessary antimicrobial exposure. Personalisation of empirical antimicrobial therapy. Role of rapid diagnostic techniques in guiding initial antimicrobial treatment.
*Begin rapid administration of crystalloids for hypotension or lactate ≥ 4 mmol/L*	Early fluid resuscitation is recommended in patients with sepsis-induced hypoperfusion. Subsequent fluid administration should be guided by fluid responsiveness and fluid tolerance. Avoidance of fluid overload is associated with improved outcomes.	Optimal initial fluid volume. Identification of patients most likely to benefit from liberal versus restrictive fluid strategies. Optimal physiological targets to guide fluid administration.
*Apply vasopressors if the patient remains hypotensive during or after fluid resuscitation*	Norepinephrine is the first-line vasopressor. Early initiation may reduce excessive fluid administration and fluid accumulation. Peripheral administration is generally safe when appropriate protocols are followed.	Optimal timing of vasopressor initiation. Personalized MAP targets. Optimal combination and sequencing of vasopressor agents.

Abbreviations: capillary refill time, CRT; mean arterial pressure, MAP; multi-drug resistant, MDR.

## Data Availability

No new data were created or analyzed in this study. Data sharing is not applicable to this article.

## Abbreviations

The following abbreviations are used in this manuscript:

AI	Artificial intelligence
ARC	Augmented renal clearance
CO_2_	Carbon Dioxide
CRT	Capillary Refill Time
DO_2_	Oxygen delivery
ICU	Intensive Care Unit
MALS	Macrophage activation-like syndrome
MAP	Mean Arterial Pressure
MDR	Multidrug-resistant
ML	Machine learning
PK/PD	Pharmacokinetics/pharmacodynamics
SSC	Surviving Sepsis Campaign
Vd	Volume of distribution
VO_2_	Oxygen consumption

## References

[1] Gray A.P., Chung E., Hsu R.L., Araki D.T., Gershberg Hayoon A., Davis Weaver N., Swetschinski L.R., Wool E.E., Han C., Mestrovic T. (2025). Global, Regional, and National Sepsis Incidence and Mortality, 1990–2021: A Systematic Analysis. Lancet Glob. Health.

[2] Prescott H.C., Antonelli M., Alhazzani W., Møller M.H., Alshamsi F., Azevedo L.C.P., Belley-Cote E., De Waele J., Derde L., Dionne J.C. (2026). Surviving Sepsis Campaign: International Guidelines for Management of Sepsis and Septic Shock 2026. Intensive Care Med..

[3] Seymour C.W., Kennedy J.N., Wang S., Chang C.-C.H., Elliott C.F., Xu Z., Berry S., Clermont G., Cooper G., Gomez H. (2019). Derivation, Validation, and Potential Treatment Implications of Novel Clinical Phenotypes for Sepsis. JAMA.

[4] Girardis M., Coloretti I., Antonelli M., Berlot G., Busani S., Cortegiani A., De Pascale G., De Rosa F.G., De Rosa S., Donadello K. (2024). Adjunctive Immunotherapeutic Agents in Patients with Sepsis and Septic Shock: A Multidisciplinary Consensus of 23. J. Anesth. Analg. Crit. Care.

[5] Evans I.V.R., Phillips G.S., Alpern E.R., Angus D.C., Friedrich M.E., Kissoon N., Lemeshow S., Levy M.M., Parker M.M., Terry K.M. (2018). Association Between the New York Sepsis Care Mandate and In-Hospital Mortality for Pediatric Sepsis. JAMA.

[6] De Pascale G., Antonelli M., Deschepper M., Arvaniti K., Blot K., Brown B.C., De Lange D., De Waele J., Dikmen Y., Dimopoulos G. (2022). Poor Timing and Failure of Source Control Are Risk Factors for Mortality in Critically Ill Patients with Secondary Peritonitis. Intensive Care Med..

[7] Cidade J.P., Póvoa P., Depuydt P., Dhaese S., De Smet K., Tabah A., Akova M., Cotta M.O., De Pascale G., Dimopoulos G. (2026). Impact of Appropriate Antimicrobial Therapy on Patient Outcomes and Antimicrobial Use: A Sub Analysis of the DIANA Study Dataset. Intensive Care Med..

[8] Seymour C.W., Gesten F., Prescott H.C., Friedrich M.E., Iwashyna T.J., Phillips G.S., Lemeshow S., Osborn T., Terry K.M., Levy M.M. (2017). Time to Treatment and Mortality during Mandated Emergency Care for Sepsis. N. Engl. J. Med..

[9] Dellinger R.P., Carlet J.M., Masur H., Gerlach H., Calandra T., Cohen J., Gea-Banacloche J., Keh D., Marshall J.C., Parker M.M. (2004). Surviving Sepsis Campaign Guidelines for Management of Severe Sepsis and Septic Shock. Intensive Care Med..

[10] Dellinger R.P., Levy M.M., Rhodes A., Annane D., Gerlach H., Opal S.M., Sevransky J.E., Sprung C.L., Douglas I.S., Jaeschke R. (2013). Surviving Sepsis Campaign: International Guidelines for Management of Severe Sepsis and Septic Shock, 2012. Intensive Care Med..

[11] Levy M.M., Evans L.E., Rhodes A. (2018). The Surviving Sepsis Campaign Bundle: 2018 Update. Crit. Care Med..

[12] Singer M., Deutschman C.S., Seymour C.W., Shankar-Hari M., Annane D., Bauer M., Bellomo R., Bernard G.R., Chiche J.-D., Coopersmith C.M. (2016). The Third International Consensus Definitions for Sepsis and Septic Shock (Sepsis-3). JAMA.

[13] Kushimoto S., Akaishi S., Sato T., Nomura R., Fujita M., Kudo D., Kawazoe Y., Yoshida Y., Miyagawa N. (2016). Lactate, a Useful Marker for Disease Mortality and Severity but an Unreliable Marker of Tissue Hypoxia/Hypoperfusion in Critically Ill Patients. Acute Med. Surg..

[14] Garcia-Alvarez M., Marik P., Bellomo R. (2014). Sepsis-Associated Hyperlactatemia. Crit. Care.

[15] Consoli A., Nurjhan N., Reilly J.J., Bier D.M., Gerich J.E. (1990). Contribution of Liver and Skeletal Muscle to Alanine and Lactate Metabolism in Humans. Am. J. Physiol.-Endocrinol. Metab..

[16] Levy B., Gibot S., Franck P., Cravoisy A., Bollaert P.-E. (2005). Relation between Muscle Na+K+ ATPase Activity and Raised Lactate Concentrations in Septic Shock: A Prospective Study. Lancet.

[17] Levy B. (2006). Lactate and Shock State: The Metabolic View. Curr. Opin. Crit. Care.

[18] Nichol A.D., Egi M., Pettila V., Bellomo R., French C., Hart G., Davies A., Stachowski E., Reade M.C., Bailey M. (2010). Relative Hyperlactatemia and Hospital Mortality in Critically Ill Patients: A Retrospective Multi-Centre Study. Crit. Care.

[19] Casserly B., Phillips G.S., Schorr C., Dellinger R.P., Townsend S.R., Osborn T.M., Reinhart K., Selvakumar N., Levy M.M. (2015). Lactate Measurements in Sepsis-Induced Tissue Hypoperfusion: Results From the Surviving Sepsis Campaign Database. Crit. Care Med..

[20] Vincent J.-L., Quintairos E Silva A., Couto L., Taccone F.S. (2016). The Value of Blood Lactate Kinetics in Critically Ill Patients: A Systematic Review. Crit. Care.

[21] Nguyen H.B., Rivers E.P., Knoblich B.P., Jacobsen G., Muzzin A., Ressler J.A., Tomlanovich M.C. (2004). Early Lactate Clearance Is Associated with Improved Outcome in Severe Sepsis and Septic Shock. Crit. Care Med..

[22] Jansen T.C., Van Bommel J., Schoonderbeek F.J., Sleeswijk Visser S.J., Van Der Klooster J.M., Lima A.P., Willemsen S.P., Bakker J. (2010). Early Lactate-Guided Therapy in Intensive Care Unit Patients: A Multicenter, Open-Label, Randomized Controlled Trial. Am. J. Respir. Crit. Care Med..

[23] Hernández G., Ospina-Tascón G.A., Damiani L.P., Estenssoro E., Dubin A., Hurtado J., Friedman G., Castro R., Alegría L., Teboul J.-L. (2019). Effect of a Resuscitation Strategy Targeting Peripheral Perfusion Status vs Serum Lactate Levels on 28-Day Mortality Among Patients With Septic Shock: The ANDROMEDA-SHOCK Randomized Clinical Trial. JAMA.

[24] Pálizas F., Lattanzio B., Durandal N., Alonso M.E., Duque J., Falcon N., Mesa R., Hunter M., Pozo M., The ANDROMEDA-SHOCK-2 Investigators for the ANDROMEDA Research Network, Spanish Society of Anesthesiology, Reanimation and Pain Therapy (SEDAR), and Latin American Intensive Care Network (LIVEN) (2025). Personalized Hemodynamic Resuscitation Targeting Capillary Refill Time in Early Septic Shock: The ANDROMEDA-SHOCK-2 Randomized Clinical Trial. JAMA.

[25] Muller G., Contou D., Ehrmann S., Martin M., Andreu P., Kamel T., Boissier F., Azais M.-A., Monnier A., Vimeux S. (2025). Deferring Arterial Catheterization in Critically Ill Patients with Shock. N. Engl. J. Med..

[26] Kreisberg R.A. (1980). Lactate Homeostasis and Lactic Acidosis. Ann. Intern. Med..

[27] Marliss E.B., Ohman J.L., Aoki T.T., Kozak G.P. (1970). Altered Redox State Obscuring Ketoacidosis in Diabetic Patients with Lactic Acidosis. N. Engl. J. Med..

[28] Sillos E.M., Shenep J.L., Burghen G.A., Pui C.-H., Behm F.G., Sandlund J.T. (2001). Lactic Acidosis: A Metabolic Complication of Hematologic Malignancies: Case Report and Review of the Literature. Cancer.

[29] Centers for Disease Control and Prevention (CDC) (1997). Lactic Acidosis Traced to Thiamine Deficiency Related to Nationwide Shortage of Multivitamins for Total Parenteral Nutrition—United States, 1997. MMWR Morb. Mortal. Wkly. Rep..

[30] Stacpoole P.W. (1997). Lactic Acidosis and Other Mitochondrial Disorders. Metabolism.

[31] Lamy B., Dargère S., Arendrup M.C., Parienti J.-J., Tattevin P. (2016). How to Optimize the Use of Blood Cultures for the Diagnosis of Bloodstream Infections? A State-of-the Art. Front. Microbiol..

[32] Vincent J.-L., Sakr Y., Singer M., Martin-Loeches I., Machado F.R., Marshall J.C., Finfer S., Pelosi P., Brazzi L., Aditianingsih D. (2020). Prevalence and Outcomes of Infection Among Patients in Intensive Care Units in 2017. JAMA.

[33] Weinstein M.P., Reller L.B., Murphy J.R., Lichtenstein K.A. (1983). The Clinical Significance of Positive Blood Cultures: A Comprehensive Analysis of 500 Episodes of Bacteremia and Fungemia in Adults. I. Laboratory and Epidemiologic Observations. Clin. Infect. Dis..

[34] Kirn T.J., Weinstein M.P. (2013). Update on Blood Cultures: How to Obtain, Process, Report, and Interpret. Clin. Microbiol. Infect..

[35] Kumar A., Roberts D., Wood K.E., Light B., Parrillo J.E., Sharma S., Suppes R., Feinstein D., Zanotti S., Taiberg L. (2006). Duration of Hypotension before Initiation of Effective Antimicrobial Therapy Is the Critical Determinant of Survival in Human Septic Shock*. Crit. Care Med..

[36] Scheer C.S., Giamarellos-Bourboulis E.J., Ferrer R., Idelevich E.A., Annane D., Artigas A., Aslan A.T., Bottari G., Bouma H.R., Černý V. (2025). Status of Sepsis Care in European Hospitals: Results from an International Cross-Sectional Survey. Am. J. Respir. Crit. Care Med..

[37] Cheng M.P., Stenstrom R., Paquette K., Stabler S.N., Akhter M., Davidson A.C., Gavric M., Lawandi A., Jinah R., Saeed Z. (2019). Blood Culture Results Before and After Antimicrobial Administration in Patients With Severe Manifestations of Sepsis: A Diagnostic Study. Ann. Intern. Med..

[38] Coburn B., Morris A.M., Tomlinson G., Detsky A.S. (2012). Does This Adult Patient With Suspected Bacteremia Require Blood Cultures?. JAMA.

[39] Posteraro B., Cortazzo V., Liotti F.M., Menchinelli G., Ippoliti C., De Angelis G., La Sorda M., Capalbo G., Vargas J., Antonelli M. (2021). Diagnosis and Treatment of Bacterial Pneumonia in Critically Ill Patients with COVID-19 Using a Multiplex PCR Assay: A Large Italian Hospital’s Five-Month Experience. Microbiol. Spectr..

[40] Timbrook T.T., Morton J.B., McConeghy K.W., Caffrey A.R., Mylonakis E., LaPlante K.L. (2017). The Effect of Molecular Rapid Diagnostic Testing on Clinical Outcomes in Bloodstream Infections: A Systematic Review and Meta-Analysis. Clin. Infect. Dis..

[41] Banerjee R., Komarow L., Li Y., Mau D., Dodd A., Geres H., Greenwood-Quaintance K., Adler A., Baliga S., Chowers M. (2026). Fast Antimicrobial Susceptibility Testing for Gram-Negative Bacteremia: The FAST Randomized Clinical Trial. JAMA.

[42] Liborio M.P., Harris P.N.A., Ravi C., Irwin A.D. (2024). Getting Up to Speed: Rapid Pathogen and Antimicrobial Resistance Diagnostics in Sepsis. Microorganisms.

[43] Virk A., Strasburg A.P., Kies K.D., Donadio A.D., Mandrekar J., Harmsen W.S., Stevens R.W., Estes L.L., Tande A.J., Challener D.W. (2024). Rapid Multiplex PCR Panel for Pneumonia in Hospitalised Patients with Suspected Pneumonia in the USA: A Single-Centre, Open-Label, Pragmatic, Randomised Controlled Trial. Lancet Microbe.

[44] Cano S., Clari M.Á., Albert E., Liñan J., Juan M., Olea B., Carbonell N., Navarro D. (2026). Protocolized Antimicrobial Stewardship Following Filmarray Pneumonia plus Panel Testing in Mechanically Ventilated Patients with Severe Lower Respiratory Tract Infection. Sci. Rep..

[45] Bassetti M., Rello J., Blasi F., Goossens H., Sotgiu G., Tavoschi L., Zasowski E.J., Arber M.R., McCool R., Patterson J.V. (2020). Systematic Review of the Impact of Appropriate versus Inappropriate Initial Antibiotic Therapy on Outcomes of Patients with Severe Bacterial Infections. Int. J. Antimicrob. Agents.

[46] Leung L.Y., Huang H.-L., Hung K.K., Leung C.Y., Lam C.C., Lo R.S., Yeung C.Y., Tsoi P.J., Lai M., Brabrand M. (2024). Door-to-Antibiotic Time and Mortality in Patients with Sepsis: Systematic Review and Meta-Analysis. Eur. J. Intern. Med..

[47] Roberts J.A., Taccone F.S., Lipman J. (2016). Understanding PK/PD. Intensive Care Med..

[48] Timsit J.-F., Ling L., De Montmollin E., Bracht H., Conway-Morris A., De Bus L., Falcone M., Harris P.N.A., Machado F.R., Paiva J.-A. (2025). Antibiotic Therapy for Severe Bacterial Infections. Intensive Care Med..

[49] Cutuli S.L., Cascarano L., Lazzaro P., Tanzarella E.S., Pintaudi G., Grieco D.L., De Pascale G., Antonelli M. (2023). Antimicrobial Exposure in Critically Ill Patients with Sepsis-Associated Multi-Organ Dysfunction Requiring Extracorporeal Organ Support: A Narrative Review. Microorganisms.

[50] Kollef M.H., Shorr A.F., Bassetti M., Timsit J.-F., Micek S.T., Michelson A.P., Garnacho-Montero J. (2021). Timing of Antibiotic Therapy in the ICU. Crit. Care.

[51] Timsit J.-F., Bassetti M., Cremer O., Daikos G., De Waele J., Kallil A., Kipnis E., Kollef M., Laupland K., Paiva J.-A. (2019). Rationalizing Antimicrobial Therapy in the ICU: A Narrative Review. Intensive Care Med..

[52] Martín-Loeches I., Leone M., Reyes L.F., Conway-Morris A., Russell L., Keat Teoh T., Rodríguez A. (2026). Precision Antibiotic Treatment in Intensive Care Unit-Acquired Lower Respiratory Tract Infections (ICU-LRTIs): Contemporary Concepts and Future Directions. Expert Rev. Anti-Infect. Ther..

[53] Cortegiani A., Russotto V., Maggiore A., Attanasio M., Naro A.R., Raineri S.M., Giarratano A. (2016). Antifungal Agents for Preventing Fungal Infections in Non-Neutropenic Critically Ill Patients. Cochrane Database Syst. Rev..

[54] Blot S.I., Taccone F.S., Van Den Abeele A.-M., Bulpa P., Meersseman W., Brusselaers N., Dimopoulos G., Paiva J.A., Misset B., Rello J. (2012). A Clinical Algorithm to Diagnose Invasive Pulmonary Aspergillosis in Critically Ill Patients. Am. J. Respir. Crit. Care Med..

[55] Thomas-Rüddel D.O., Schlattmann P., Pletz M., Kurzai O., Bloos F. (2022). Risk Factors for Invasive Candida Infection in Critically Ill Patients. CHEST.

[56] Ippolito M., Cortegiani A. (2023). Empirical Decision-Making for Antimicrobial Therapy in Critically Ill Patients. BJA Educ..

[57] Niederman M.S., Baron R.M., Bouadma L., Calandra T., Daneman N., DeWaele J., Kollef M.H., Lipman J., Nair G.B. (2021). Initial Antimicrobial Management of Sepsis. Crit. Care.

[58] Evans L., Rhodes A., Alhazzani W., Antonelli M., Coopersmith C.M., French C., Machado F.R., Mcintyre L., Ostermann M., Prescott H.C. (2021). Surviving Sepsis Campaign: International Guidelines for Management of Sepsis and Septic Shock 2021. Intensive Care Med..

[59] Kuttab H.I., Evans C.G., Lykins J.D., Hughes M.D., Kopec J.A., Hernandez M.A., Ward M.A. (2023). The Effect of Fluid Resuscitation Timing in Early Sepsis Resuscitation. J. Intensive Care Med..

[60] Piehl M., Adejumo F.F., Maio V.D. (2025). The Association Between Time to Completion of at Least 30 mL/Kg and Hospital Outcomes Among Patients With Septic Shock. Crit. Care Explor..

[61] Meyhoff T.S., Hjortrup P.B., Wetterslev J., Sivapalan P., Laake J.H., Cronhjort M., Jakob S.M., Cecconi M., Nalos M., Ostermann M. (2022). Restriction of Intravenous Fluid in ICU Patients with Septic Shock. N. Engl. J. Med..

[62] Shapiro N.I., Douglas I.S., Brower R.G., Brown S.M., Exline M.C., Ginde A.A., Gong M.N., Grissom C.K., Hayden D., The National Heart, Lung, and Blood Institute Prevention and Early Treatment of Acute Lung Injury Clinical Trials Network (2023). Early Restrictive or Liberal Fluid Management for Sepsis-Induced Hypotension. N. Engl. J. Med..

[63] Marik P.E., Cavallazzi R. (2013). Does the Central Venous Pressure Predict Fluid Responsiveness? An Updated Meta-Analysis and a Plea for Some Common Sense. Crit. Care Med..

[64] Monnet X., Marik P., Teboul J.-L. (2016). Passive Leg Raising for Predicting Fluid Responsiveness: A Systematic Review and Meta-Analysis. Intensive Care Med..

[65] Biais M., De Courson H., Lanchon R., Pereira B., Bardonneau G., Griton M., Sesay M., Nouette-Gaulain K. (2017). Mini-Fluid Challenge of 100 mL of Crystalloid Predicts Fluid Responsiveness in the Operating Room. Anesthesiology.

[66] Monnet X., Shi R., Teboul J.-L. (2022). Prediction of Fluid Responsiveness. What’s New?. Ann. Intensive Care.

[67] Monnet X., Julien F., Ait-Hamou N., Lequoy M., Gosset C., Jozwiak M., Persichini R., Anguel N., Richard C., Teboul J.-L. (2013). Lactate and Venoarterial Carbon Dioxide Difference/Arterial-Venous Oxygen Difference Ratio, but Not Central Venous Oxygen Saturation, Predict Increase in Oxygen Consumption in Fluid Responders*. Crit. Care Med..

[68] Monnet X., Lai C., Teboul J.-L. (2023). How I Personalize Fluid Therapy in Septic Shock?. Crit. Care.

[69] Malbrain M.L.N.G., Van Regenmortel N., Saugel B., De Tavernier B., Van Gaal P.-J., Joannes-Boyau O., Teboul J.-L., Rice T.W., Mythen M., Monnet X. (2018). Principles of Fluid Management and Stewardship in Septic Shock: It Is Time to Consider the Four D’s and the Four Phases of Fluid Therapy. Ann. Intensive Care.

[70] Messmer A.S., Zingg C., Müller M., Gerber J.L., Schefold J.C., Pfortmueller C.A. (2020). Fluid Overload and Mortality in Adult Critical Care Patients—A Systematic Review and Meta-Analysis of Observational Studies*. Crit. Care Med..

[71] Messina A., Albini M., Samuelli N., Brunati A., Costantini E., Lionetti G., Lubian M., Greco M., Matronola G.M., Piccirillo F. (2024). Fluid Boluses and Infusions in the Early Phase of Resuscitation from Septic Shock and Sepsis-Induced Hypotension: A Retrospective Report and Outcome Analysis from a Tertiary Hospital. Ann. Intensive Care.

[72] Monnet X., Messina A., Greco M., Bakker J., Aissaoui N., Cecconi M., Coppalini G., De Backer D., Edul V.K., Evans L. (2025). ESICM Guidelines on Circulatory Shock and Hemodynamic Monitoring 2025. Intensive Care Med..

[73] Fage N., Chew M.S., Asfar P. (2026). Vasopressor Use in Septic Shock: Separating Evidence, Practice, and Future Directions. Intensive Care Med..

[74] Carelli S., Grieco D.L., Castaldo F., Morciano D.A., Bongiovanni F., Cisterna I., De Pascale G., Antonelli M., Dell’Anna A.M. (2026). Effect of Norepinephrine on Ventricular Systolic Function during Septic Shock: The ENESySS Study. J. Anesth. Analg. Crit. Care.

[75] Permpikul C., Tongyoo S., Viarasilpa T., Trainarongsakul T., Chakorn T., Udompanturak S. (2019). Early Use of Norepinephrine in Septic Shock Resuscitation (CENSER). A Randomized Trial. Am. J. Respir. Crit. Care Med..

[76] Ospina-Tascón G.A., Hernandez G., Alvarez I., Calderón-Tapia L.E., Manzano-Nunez R., Sánchez-Ortiz A.I., Quiñones E., Ruiz-Yucuma J.E., Aldana J.L., Teboul J.-L. (2020). Effects of Very Early Start of Norepinephrine in Patients with Septic Shock: A Propensity Score-Based Analysis. Crit. Care.

[77] Peake S.L., Macdonald S.P.J., Howe B.D., Milford E., Jones P., Arendts G., Bailey M., Burcham J., Delaney A., ARISE FLUIDS Investigators, the ANZICS Clinical Trials Group, and the ACEM Clinical Trials Network (2026). Vasopressors or Fluids in Early Septic Shock. N. Engl. J. Med..

[78] Owen V.S., Rosgen B.K., Cherak S.J., Ferland A., Stelfox H.T., Fiest K.M., Niven D.J. (2021). Adverse Events Associated with Administration of Vasopressor Medications through a Peripheral Intravenous Catheter: A Systematic Review and Meta-Analysis. Crit. Care.

[79] Asfar P., Meziani F., Hamel J.-F., Grelon F., Megarbane B., Anguel N., Mira J.-P., Dequin P.-F., Gergaud S., Weiss N. (2014). High versus Low Blood-Pressure Target in Patients with Septic Shock. N. Engl. J. Med..

[80] Lamontagne F., Richards-Belle A., Thomas K., Harrison D.A., Sadique M.Z., Grieve R.D., Camsooksai J., Darnell R., Gordon A.C., Henry D. (2020). Effect of Reduced Exposure to Vasopressors on 90-Day Mortality in Older Critically Ill Patients With Vasodilatory Hypotension: A Randomized Clinical Trial. JAMA.

[81] Russell J.A., Walley K.R., Singer J., Gordon A.C., Hébert P.C., Cooper D.J., Holmes C.L., Mehta S., Granton J.T., Storms M.M. (2008). Vasopressin versus Norepinephrine Infusion in Patients with Septic Shock. N. Engl. J. Med..

[82] Gordon A.C., Mason A.J., Thirunavukkarasu N., Perkins G.D., Cecconi M., Cepkova M., Pogson D.G., Aya H.D., Anjum A., Frazier G.J. (2016). Effect of Early Vasopressin vs Norepinephrine on Kidney Failure in Patients With Septic Shock: The VANISH Randomized Clinical Trial. JAMA.

[83] Khanna A., English S.W., Wang X.S., Ham K., Tumlin J., Szerlip H., Busse L.W., Altaweel L., Albertson T.E., Mackey C. (2017). Angiotensin II for the Treatment of Vasodilatory Shock. N. Engl. J. Med..

[84] Umemura Y., Abe T., Ogura H., Fujishima S., Kushimoto S., Shiraishi A., Saitoh D., Mayumi T., Otomo Y., Hifumi T. (2022). Hour-1 Bundle Adherence Was Associated with Reduction of in-Hospital Mortality among Patients with Sepsis in Japan. PLoS ONE.

[85] Rhee C., Train S.E., Filbin M.R., Park S.T., Mohr N.M., Zepeski A., Faine B.A., Roach D.J., Porter E., Shappell C.N. (2025). Complex Sepsis Presentations, SEP-1 Compliance, and Outcomes. JAMA Netw. Open.

[86] Bolanaki M., Kurland L., Brabrand M., Daniels R., Govender K., Hanses F., Innocenti F., Lassen A., Martin-Loeches I., Möckel M. (2025). Current Sepsis Management Practices in European Emergency Departments: The ISG-Emergency Department European Survey. Eur. J. Emerg. Med..

[87] You J.S., Park Y.S., Chung S.P., Lee H.S., Jeon S., Kim W.Y., Shin T.G., Jo Y.H., Kang G.H., Choi S.H. (2022). Relationship between Time of Emergency Department Admission and Adherence to the Surviving Sepsis Campaign Bundle in Patients with Septic Shock. Crit. Care.

[88] Sasmito P., Pranata S., Pamungkas R.A., Emaliyawati E., Arifani N. (2024). Challenges of Implementing the Hour-1 Sepsis Bundle: A Qualitative Study from a Secondary Hospital in Indonesia. Acute Crit. Care.

[89] Charoentanyarak S., Chopetgool T., Boonsawat W., Sawanyawisuth K. (2026). Completion of the 1-Hour Sepsis Bundle on 90-Day Mortality in Adult Patients With Sepsis. JACEP Open.

[90] Ko B.S., Choi S.-H., Shin T.G., Kim K., Jo Y.H., Ryoo S.M., Park Y.S., Kwon W.Y., Choi H.S., Chung S.P. (2021). Impact of 1-Hour Bundle Achievement in Septic Shock. J. Clin. Med..

[91] Freund Y., Cancella De Abreu M., Lebal S., Rousseau A., Lafon T., Yordanov Y., Macrez R., Coisy F., Le Borgne P., Femy F. (2024). Effect of the 1-h Bundle on Mortality in Patients with Suspected Sepsis in the Emergency Department: A Stepped Wedge Cluster Randomized Clinical Trial. Intensive Care Med..

[92] Hu B., Xiang H., Dong Y., Portner E., Peng Z., Kashani K. (2020). Timeline of Sepsis Bundle Component Completion and Its Association with Septic Shock Outcomes. J. Crit. Care.

[93] De Pascale G., Carelli S., Vallecoccia M.S., Cutuli S.L., Taccheri T., Montini L., Bello G., Spanu T., Tumbarello M., Cicchetti A. (2019). Risk Factors for Mortality and Cost Implications of Complicated Intra-Abdominal Infections in Critically Ill Patients. J. Crit. Care.

[94] Martínez M.L., Ferrer R., Torrents E., Guillamat-Prats R., Gomà G., Suárez D., Álvarez-Rocha L., Pozo Laderas J.C., Martín-Loeches I., Levy M.M. (2017). Impact of Source Control in Patients With Severe Sepsis and Septic Shock*. Crit. Care Med..

[95] Slim M.A., Van Mourik N., Bakkerus L., Fuller K., Acharya L., Giannidis T., Dionne J.C., Oczkowski S.J.W., Netea M.G., Pickkers P. (2024). Towards Personalized Medicine: A Scoping Review of Immunotherapy in Sepsis. Crit. Care.

[96] Leventogiannis K., Kyriazopoulou E., Antonakos N., Kotsaki A., Tsangaris I., Markopoulou D., Grondman I., Rovina N., Theodorou V., Antoniadou E. (2022). Toward Personalized Immunotherapy in Sepsis: The PROVIDE Randomized Clinical Trial. Cell Rep. Med..

[97] Giamarellos-Bourboulis E.J., Kotsaki A., Kotsamidi I., Efthymiou A., Koutsoukou V., Ehler J., Paridou A., Frantzeskaki F., Müller M.C.A., Pickkers P. (2026). Precision Immunotherapy to Improve Sepsis Outcomes: The ImmunoSep Randomized Clinical Trial. JAMA.

[98] Kalimouttou A., Lerner I., Cheurfa C., Jannot A.-S., Pirracchio R. (2023). Machine-Learning-Derived Sepsis Bundle of Care. Intensive Care Med..

[99] Li F., Wang S., Gao Z., Qing M., Pan S., Liu Y., Hu C. (2025). Harnessing Artificial Intelligence in Sepsis Care: Advances in Early Detection, Personalized Treatment, and Real-Time Monitoring. Front. Med..

[100] Zhang Z., Chen L., Xu P., Wang Q., Zhang J., Chen K., Clements C.M., Celi L.A., Herasevich V., Hong Y. (2022). Effectiveness of Automated Alerting System Compared to Usual Care for the Management of Sepsis. npj Digit. Med..

